# Supplementary material for: Early treatment regimens achieve sustained virologic remission in infant macaques infected with SIV at birth
Source: Nat Commun. 2022 Aug 16;13:4823. doi: 10.1038/s41467-022-32554-z (PMC9381774; doi:10.1038/s41467-022-32554-z)
Supplement: Supplementary file 1 — Supplementary Information [file 41467_2022_32554_MOESM1_ESM.pdf]

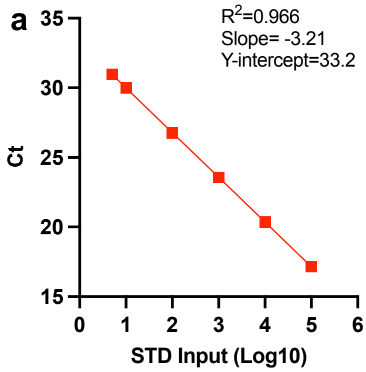


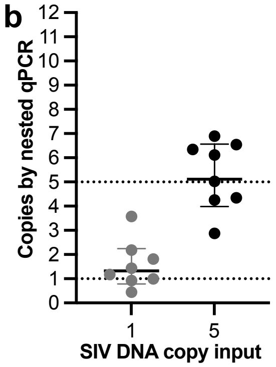


**Low copies of total SIV DNA by nested qPCR**

| Spiked SIV DNA (copies/reaction) | LTR U5 (n=8) |
|---|---|
| | Mean SD |
| 1 | 1.57±0.97 |
| 5 | 5.56±1.52 |

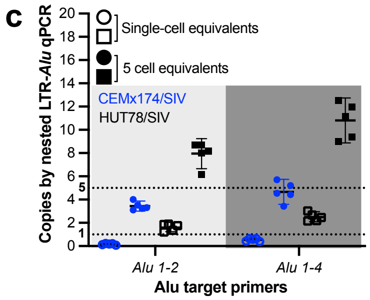


**Low copies of integrated SIV DNA by nested LTR-Alu qPCR**

| Cell number (genomic DNA per reaction) | Nested LTR-Alu qPCR (n=5) | | | |
|---|---|---|---|---|
| | CEM×174/SIV | | HUT78/SIV | |
| | Alu 1-2 | Alu 1-4 | Alu 1-2 | Alu 1-4 |
| | Mean SD | Mean SD | Mean SD | Mean SD |
| 1 | 0.16±0.06 | 0.91±0.07 | 0.53±0.12 | 2.5±0.36 |
| 5 | 3.44±0.35 | 4.6±0.86 | 7.95±1.04 | 10.8±1.56 |

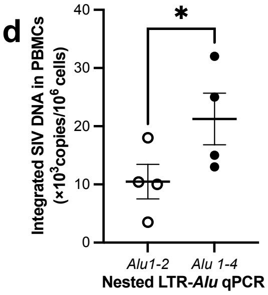


**Integrated SIV DNA in PBMCs**

| Alu primers (Nested LTR-Alu qPCR) | Animal ID | | | |
|---|---|---|---|---|
| | LC20 | LD05 | LG30 | LH01 |
| Alu 1-2 | 1.0E+04 | 1.1E+04 | 3.5E+03 | 1.8E+04 |
| Alu 1-4 | 2.5E+04 | 1.5E+04 | 1.3E+04 | 3.2E+04 |

**Supplementary Figure 1. Sensitivity of nested (LTR-Alu) qPCR in the measurement of the lower SIV DNA/proviral DNA input using SIV DNA/integrated proviral DNA standard.** (**a**) Linearity of standard by nested (long terminal repeat/LTR U5 region-Alu) qPCR. Y and X-axis represent the threshold cycle (Ct) and the starting quantity of standard, respectively. $\boldsymbol{y}$=-3.21$\boldsymbol{x}$+33.2. (**b**) Sensitivity and reproducibility of nested qPCR to detect one or five copies of SIV plasmid DNA standard. Data are presented as the mean ± SD of the 8 technically independent experiments (Mean with 95% CI). SIV DNA standards were spiked with cellular genomic DNA extracted from SIV naïve PBMCs ($1\times10^5$ cell equivalents). (**c**) Sensitivity of nested LTR-Alu qPCR to detect integrated SIV DNA in genomic DNA from CEMx174 versus HUT78 cell line. Data are presented as the mean ± SD of the 5 technically independent experiments. CEMx174 (ARP-13239) and HUT78/SIV (ARP-160) cell lines (NIH AIDS Reagent Program) were cultured for genomic DNA extraction, in which genomic DNA equivalent to cell numbers was determined by human/NHP RPPR30-based ddPCR (PMID: 29483619). (**d**) Levels of integrated SIV DNA in PBMC samples from chronically SIV-infected rhesus macaques (n=4), measured by nested Alu qPCR. Data are presented as the scatter plot from 4 individual adult animals, with median value. *, P value (0.02) was determined by paired t test. Source data are provided as a Source Data file.

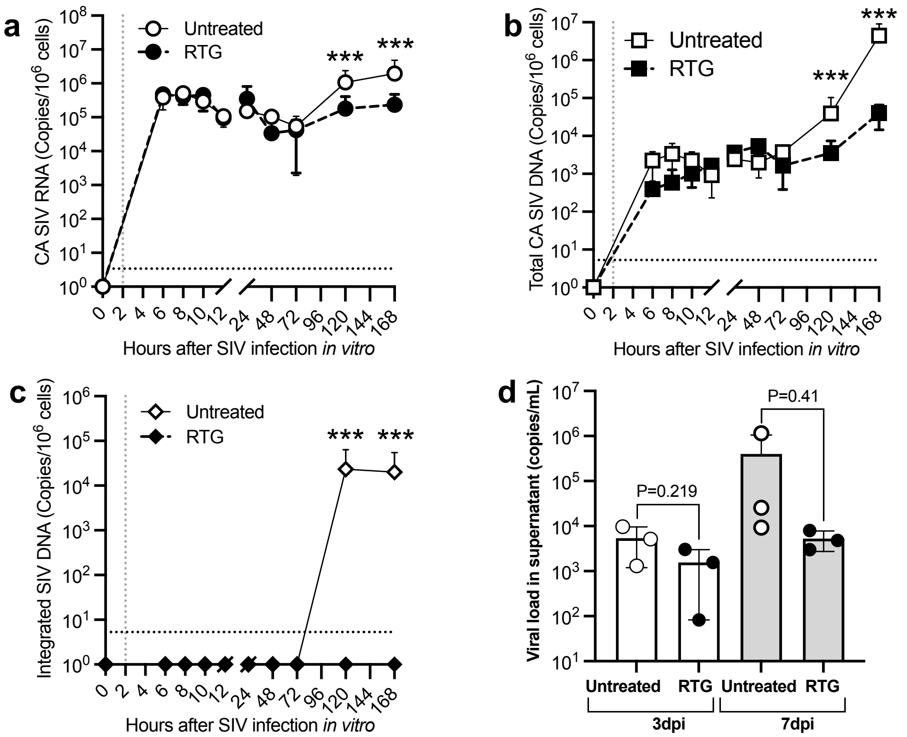


**Supplementary Figure 2. Viral parameters in infant PBMCs infected with SIV in vitro, in presence or absence integrase inhibitor**. Dynamic levels of cell-associated total SIV RNA (**a**), total SIV DNA (**b**) and integrated proviral DNA (**c**) in PBMCs infected with SIV in vitro, with or without raltegravir (RTG) treatment at 2h after SIV infection. Data are presented as the mean ± SD of technical duplicates, representative of two independent experiments. ***, $P<0.001$, compared with untreated control at the same timepoint by paired t test analysis. (**d**) Viral load in supernatants at day 3 and 7 post SIV infection, in presence or absence of RTG. Data are presented as box and scatter plot with bar, with the mean ± SD of minimum and maximum values, representative of two independent experiments. P values are determined with two-tailed t-test. Cryopreserved PBMCs from uninfected one-month-old infants (n=3) were recovered overnight, followed by cell activation and SIV infection. Cells were treated by RTG (final concentration, 1.0μM) after 2 hours of SIV incubation. Cell pellets and supernatants were harvested for viral RNA/DNA or viral load measurement at time scheduled. Source data are provided as a Source Data file.

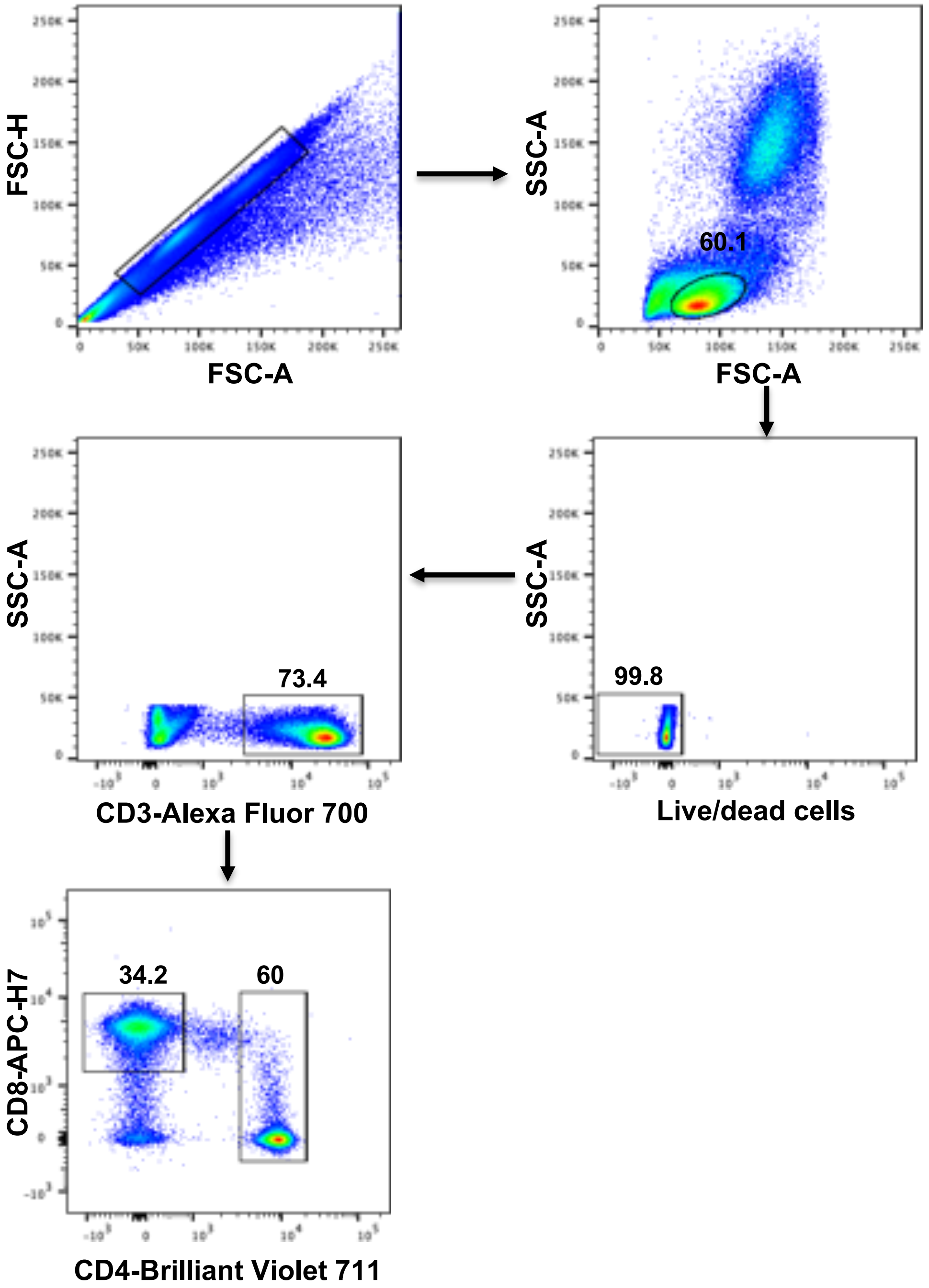


**Supplementary Figure 3. Gate strategy of peripheral CD4+ T cells in the study.**

**Supplementary Table 1. Primers and hydrolysis probes sequences used in the study.**

| Target | Oligonucleotide | Sequence ('5 to 3') | Amplicon length |
|---|---|---|---|
| **Rh CCR5** | Forward primer | CCAGAAGAGCTGCGACATCC | |
| | Reverse primer | CTAATAGGCCAAGCAGCTGAGG | 75bp |
| | Probe | (MAX)-TTCCCCTAC/ZEN/AAGAAACTCTCCCCGGTAAGTA-(IBFQ) | |
| **SIV gag** | Forward primer | GCAGAGGAGGAAATTACCCAGTAC | |
| | Reverse primer | CAATTTTACCCAGGCATTTAATGTT | 84bp |
| | Probe | (FAM)-TGTCCACCT/ZEN/GCCATTAAGCCCGA-(BHQ-1) | |
| **SIV LTR U5** | Forward primer | AGGCTGGCAGATTGAGCCCTGGGAGGTTC | |
| | Reverse primer | CCAGGCGGCGACTAGGAGAGATGGGAACAC | 200bp |
| | Probe | (FAM)-TTCCCTGCT/ZEN/AGACTCTCACCAGCACTTGG-(BHQ-1) | |
| **Alu** | Alu-1 | CTGTAATCCTAGTACTTTGGGAGGC | |
| | Alu-2 | CTCCTGCCTCAGCCTCCTGAGTA | |
| | Alu-3 | TAATCCCAGCACTTTGGGAGGC | |
| | | TAATCTCATCACTTTGGGAGGC | |
| | | TAATCCCAGGACTTTCGGAGGC | |
| | | TAATCCTAGCACTTTGGGAGGC | |
| | | TAAATCCAGCACTTTGGGAGGC | |
| | Alu-4 | ATTAGGGTCGTGAAACCCTCCG | |
| | | ATTAGGATCGTGAAACCCTCCG | |
| | | ATTAGGGTCCTGAAAGCCTCCG | |
| | | ATTTAGGTCGTGAAACCCTCCG | |
| | | ATTAGAGTAGTGAAACCCTCCG | |

**Supplementary Table 2. Neonatal animals and plasma viral load post SIV inoculation at birth.**

| Animal | 1dpi | 2dpi | 3dpi | 4dpi | 5dpi | 7dpi | Sampling |
|---|---|---|---|---|---|---|---|
| PC64 | 8.30E+01 | | | | | | |
| PC67 | 8.30E+01 | | | | | | |
| PD43 | 1.09E+03 | | | | | | 1dpi necropsy |
| PE37 | 3.83E+03 | | | | | | 11dpi necropsy |
| PF28 | 2.35E+03 | | | | | | 28dpi necropsy |
| PE34 | 8.30E+01 | | | | | | Before treatment |
| PC58 | | 3.15E+03 | | | | | |
| PC92 | | 9.20E+02 | | | | | |
| PD51 | | 1.82E+03 | | | | | 2dpi necropsy |
| PC83 | | | 1.62E+04 | | | | |
| PC84 | | | 3.58E+04 | | | | |
| PD44 | | | 1.33E+04 | | | | |
| HM62 | | | 1.19E+04 | | | | |
| HN61 | | | 1.29E+03 | | | | 3dpi necropsy |
| NP36 | | | 1.88E+04 | | | | 28dpi necropsy |
| PE71 | | | 1.36E+05 | | | | Before treatment |
| NP27 | | | 2.63E+03 | | | | Before treatment |
| PD32 | | | | 5.13E+05 | | | |
| PD34 | | | | 1.54E+06 | | | Before treatment |
| PC32 | | | | | 1.87E+07 | | |
| PD03 | | | | | 1.04E+07 | | 5dpi necropsy |
| PE46 | | | | | 7.71E+06 | | Before treatment |
| PB96 | | | | | | 4.80E+07 | 7dpi necropsy |

**Supplementary Table 3. MHC I alleles in the five experimental infants.** None of these infants had protective MHC class I alleles (Mamu-B*08, Mamu-A*01, or Mamu-B*17).

| Animal ID | A*01 | A*02 | A*08 | A*11 | B*01 | B*03 | B*04 | B*08 | B*17 | DRB*w201 |
|---|---|---|---|---|---|---|---|---|---|---|
| POS | + | + | + | + | + | + | + | + | + | + |
| NEG | − | − | − | − | − | − | − | − | − | − |
| NG22 | − | + | − | − | − | − | − | − | − | − |
| NG24 | − | + | + | − | + | − | − | − | − | − |
| NG90 | − | + | − | − | + | − | − | − | − | + |
| NR90 | − | − | + | − | − | − | − | − | − | − |
| NT17 | − | + | + | − | − | − | − | − | − | + |
