# Supplementary material for: Early treatment regimens achieve sustained virologic remission in infant macaques infected with SIV at birth
Source: Nat Commun. 2022 Aug 16;13:4823. doi: 10.1038/s41467-022-32554-z (PMC9381774; doi:10.1038/s41467-022-32554-z)
Supplement: Supplementary file 3 — Reporting Summary [file 41467_2022_32554_MOESM3_ESM.pdf]

Corresponding author(s): Dr. Huanbin Xu

Last updated by author(s): Jul 25, 2022

Reporting Summary

Nature Portfolio wishes to improve the reproducibility of the work that we publish. This form provides structure for consistency and transparency in reporting. For further information on Nature Portfolio policies, see our Editorial Policies and the Editorial Policy Checklist.

Statistics

For all statistical analyses, confirm that the following items are present in the figure legend, table legend, main text, or Methods section.

n/a	Confirmed	
☐	☒	The exact sample size (*n*) for each experimental group/condition, given as a discrete number and unit of measurement
☐	☒	A statement on whether measurements were taken from distinct samples or whether the same sample was measured repeatedly
☐	☒	The statistical test(s) used AND whether they are one- or two-sided<br>*Only common tests should be described solely by name; describe more complex techniques in the Methods section.*
☒	☐	A description of all covariates tested
☒	☐	A description of any assumptions or corrections, such as tests of normality and adjustment for multiple comparisons
☐	☒	A full description of the statistical parameters including central tendency (e.g. means) or other basic estimates (e.g. regression coefficient) AND variation (e.g. standard deviation) or associated estimates of uncertainty (e.g. confidence intervals)
☐	☒	For null hypothesis testing, the test statistic (e.g. *F*, *t*, *r*) with confidence intervals, effect sizes, degrees of freedom and *P* value noted<br>*Give P values as exact values whenever suitable.*
☒	☐	For Bayesian analysis, information on the choice of priors and Markov chain Monte Carlo settings
☒	☐	For hierarchical and complex designs, identification of the appropriate level for tests and full reporting of outcomes
☐	☒	Estimates of effect sizes (e.g. Cohen's *d*, Pearson's *r*), indicating how they were calculated

Our web collection on statistics for biologists contains articles on many of the points above.

Software and code

Policy information about availability of computer code

Data collection	qPCR, flow cytometry, ELISA, neutralizing antibody assay.
Data analysis	Statistical analyses were performed by GraphPad Prism 9.0 Software (GraphPad Software, San Diego, CA). Statistical comparison between groups at different time points was analyzed using Mann-Whitney test or two-tailed t-test. A nominal α level of 0.05 was used to define statistical significance and the data are presented as mean +/- SEM or SD. The relationship of integrated viral reservoir size in tissues with the timing after viral inoculation was analyzed using a nonlinear regression curve fitting model. QuantaSoft™ Software (Regulatory Edition #1864011, Bio-Rad) were used in ddPCR. FlowJo software (Version 10.8.1, Tree Star, Ashland, OR) for flow cytometry analysis. The ELISA and nAb assays were calculated by standard curve Microsoft Excel.

For manuscripts utilizing custom algorithms or software that are central to the research but not yet described in published literature, software must be made available to editors and reviewers. We strongly encourage code deposition in a community repository (e.g. GitHub). See the Nature Portfolio guidelines for submitting code & software for further information.

Data

Policy information about availability of data

All manuscripts must include a data availability statement. This statement should provide the following information, where applicable:

- Accession codes, unique identifiers, or web links for publicly available datasets
- A description of any restrictions on data availability
- For clinical datasets or third party data, please ensure that the statement adheres to our policy

Source data are provided with this paper in the Source Data file and Supplementary Information.

Field-specific reporting

Please select the one below that is the best fit for your research. If you are not sure, read the appropriate sections before making your selection.

☒ Life sciences ☐ Behavioural & social sciences ☐ Ecological, evolutionary & environmental sciences

For a reference copy of the document with all sections, see nature.com/documents/nr-reporting-summary-flat.pdf

Life sciences study design

All studies must disclose on these points even when the disclosure is negative.

Sample size	A total of 31 newborn, Indian-origin rhesus macaques (Macaca mulatta) were utilized in this study. 14 neonatal macaques were euthanized for complete tissue collection at day 1 (n=3), 2 (n=3), 3 (n=5), 5 (n=2) and 7 (n=1) post identical SIV inoculation within 6h after birth. An additional 12 infant macaques received combined antiretroviral treatment (cART) with integrase inhibitor (TFV/FTC/DGT) initiated at day 3 (n=3), 4 (n=3) or 5 (n=3), or untreated (n=3) post SIV infection for 28 days. Staggered early blood sampling (1.2cc EDTA blood; newborns ~450g) was collected from untreated animal groups at 28-day SIV infection or before treatment. For a long-term intervention, 5 additional animals were infected and treated with cART initiated on day 3 and continued daily for 9 months. Previous plasma and PBMC data from age-matched SIV-infected infant macaques (n=9), which were infected with the identical SIV lot, dose, and route were used to compare plasma viral load and the percentages of peripheral CD4+ T cells. Data were presented as scatter plot (Figs. 1b-1h, 2a-2c) and box (Figs. 2e-2g) for individual infant macaques, with median value or the mean ± SEM of viral parameters. Statistical significances were analyzed by a Mann-Whitney test or two-tailed t-test. Correlation of proviral reservoir seeding with the timing after SIV infection in tissues was statistically evaluated by curve fitting using nonlinear regression (Figs. 1i-1m). Another data represented the mean ± SEM of plasma viral loads from each group for 28 days treatment, and p value was determined by two-tailed t-test at the different time points (Fig.2d, n=3 each group for total 4 groups). In Figs. 3a-3h, data were presented as the mean ± SEM of peripheral CD4+ T cell percentage and viral RNA/DNA copies in control group at designated time points, compared with those in individual experimental animal cohorts. For the Ab assay (Figs. 3k and 3l) and supplementary data (Suppl Figs. 1 and 2), data were presented as the mean ± SD of technical duplicates, representative of two or more independent experiments. P values were determined with two-tailed t-test or paired t-test. All samples collected in the study were used to statistical analysis, which are sufficient (n>/=3 at critical time point) to address the consistence and statistical significance.
Data exclusions	No data exclusions for statistical analysis.
Replication	Data, determined by qPCR , were generated with duplicates, or represented at least two (ELISA) or more (technical replication of supplementary data) independent experiments.
Randomization	Infant macaques with gender difference were randomly assigned for the study.
Blinding	Data were collected by a double-blind measurements. Investigators were blinded to perform experiments to group allocation including core service, veterinary animal treatment and lab research.

Reporting for specific materials, systems and methods

We require information from authors about some types of materials, experimental systems and methods used in many studies. Here, indicate whether each material, system or method listed is relevant to your study. If you are not sure if a list item applies to your research, read the appropriate section before selecting a response.

Materials & experimental systems

n/a	Involved in the study
☐	☒ Antibodies
☐	☒ Eukaryotic cell lines
☒	☐ Palaeontology and archaeology
☐	☒ Animals and other organisms
☒	☐ Human research participants
☒	☐ Clinical data
☒	☐ Dual use research of concern

Methods

n/a	Involved in the study
☒	☐ ChIP-seq
☐	☒ Flow cytometry
☒	☐ MRI-based neuroimaging

Antibodies

Antibodies used	Antibodies used in this study: CD3-Alexa Fluor 700 (clone SP34-2, Cat No: 557917, Lot No: 0293255, BD Biosciences), CD4-Brilliant Violet 711 (clone OKT4, Cat No: 317440, Lot No: B293041, BioLegend), CD8-APC-H7 (clone SK1, Cat No: 560179, Lot No: 1039448, BD Biosciences), CD8-PE (clone RPA-T8, Cat No: 555367, BD Biosciences), and 1:1000 diluted HRP-conjugated goat anti-human IgA+IgG +IgM (Cat No: 109-035-064, Jackson ImmunoResearch Laboratories, Inc., West Grove, PA).
Validation	Antibodies used in phenotyping and ELISA are shown cross-reactivity with rhesus macaque species utilized according to manufacturer's websites and our long-term studies. Every effort is made to purchase antibodies in sufficient quantities at one time

and properly stored to complete a study to avoid variation. Should it become necessary to purchase additional reagents, we made every effort to use the same vendor and company, avoiding bias introduced by any batch-to-batch variation in reagents.

Eukaryotic cell lines

Policy information about cell lines

Cell line source(s)	CEMx174 (ARP-13239) and HUT78/SIV (ARP-160) cell lines (NIH AIDS Reagent Program)
Authentication	Both cell lines are authenticated, and cell cultures are followed by instructions from NIH AIDS Reagent Program.
Mycoplasma contamination	Not tested.
Commonly misidentified lines (See ICLAC register)	None

Animals and other organisms

Policy information about studies involving animals; ARRIVE guidelines recommended for reporting animal research

Laboratory animals	Male and Female indian newborn rhesus macaques (Macaca mulatta) after birth (age 0) were randomly assigned and utilized in the study.
Wild animals	No wild animals were used in the study.
Field-collected samples	No field-collected samples were used in the study.
Ethics oversight	All animals in this study were housed at the Tulane National Primate Research Center in accordance with the Association for Assessment and Accreditation of Laboratory Animal Care International standards. All studies were reviewed and approved by the Tulane University Institutional Animal Care and Use Committee under protocol number P0401. Animal housing and studies were carried out in strict accordance with the recommendations in the Guide for the Care and Use of Laboratory Animals of the National Institutes of Health (NIH, AAALAC #000594) and with the recommendations of the Weather all report, "The Use of Non-Human Primates in Research". All procedures were performed under anesthesia using ketamine, All efforts were made to minimize stress, improve housing conditions, and to provide enrichment opportunities .

Note that full information on the approval of the study protocol must also be provided in the manuscript.

Flow Cytometry

Plots

Confirm that:

- [x] The axis labels state the marker and fluorochrome used (e.g. CD4-FITC).
- [x] The axis scales are clearly visible. Include numbers along axes only for bottom left plot of group (a 'group' is an analysis of identical markers).
- [ ] All plots are contour plots with outliers or pseudocolor plots.
- [x] A numerical value for number of cells or percentage (with statistics) is provided.

Methodology

Sample preparation	Fresh lymphocytes were isolated from EDTA-treated blood by density gradient centrifugation, or from lymphoid tissues in our routine lab protocol.
Instrument	FACS FORTESSA (Becton Dickinson, San Jose, CA).
Software	FlowJo software (Tree Star, Ashland, OR).
Cell population abundance	At least 1 million cells per sample acquired by Flow cytometers.
Gating strategy	CD4+ and CD8+ T cells gated CD3+ T cells.

- [x] Tick this box to confirm that a figure exemplifying the gating strategy is provided in the Supplementary Information.

March 2021
